# Coronary Stents: History, Design, and Construction

**DOI:** 10.3390/jcm7060126

**Published:** 2018-05-29

**Authors:** Torrey Schmidt, J. Dawn Abbott

**Affiliations:** Department of Medicine, Division of Cardiology, Rhode Island Hospital, Brown Medical School, Providence, RI 02903, USA; torrey.schmidt@lifespan.org

**Keywords:** stents, percutaneous coronary intervention, drug elution, polymer

## Abstract

The history of percutaneous coronary intervention (PCI) is marked by rapid technological advancements that have taken place over the past 40 years. After a period of balloon angioplasty, which was marred by risk of abrupt vessel closure and vessel recoil, balloon expandable metal alloy stents were the mainstay of PCI. The introduction of drug eluting stents (DES) targeted in-stent restenosis, a common mode of stent failure, and ushered in the current PCI era. Since the first generation of DES, advances in polymer science and stent design have advanced the field. The current generation of DES has thin struts, are highly deliverable, have biocompatible or absorbable polymers, and outstanding safety and efficacy profiles. In this review, we discuss the technological advancements in stent development, design, and construction, with an emphasis on balloon expandable stents. The aspects of stent properties, metal alloys, bioresorbable vascular scaffolds, drug elution, and polymers will be covered.

## 1. Introduction

Cardiovascular disease is highly prevalent, affecting an estimated 92.1 million people in the United States in 2017 [1,2]. Revascularization with percutaneous coronary intervention (PCI) is an effective therapy for reducing angina in stable ischemic heart disease and major adverse cardiac events in acute coronary syndromes [3]. The rapid evolution of PCI is one of the technological marvels of this century. Prior to the seminal work of Dr. Gruentzig in 1978, the only mode of coronary revascularization was surgical bypass grafting [4]. The field has continually evolved, since the first coronary angioplasty was performed to relieve angina in a human, using a primitive balloon dilation catheter [4]. In this review, we discuss the technological advancements in PCI over the past 40 years, with an emphasis on stent development, design, and construction.

**Figure 1:**
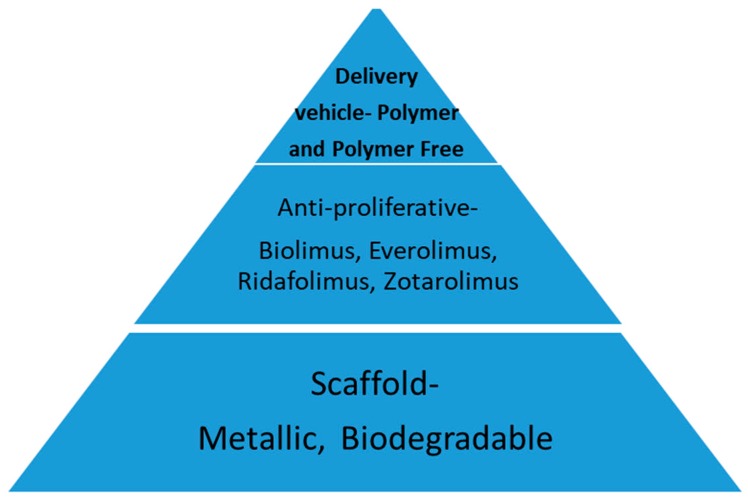
Schematic of components of coronary stent. Coronary stents are composed of a scaffold, delivery vehicle for anti-proliferative agent, and an anti-proliferative agent.

The initial technique for percutaneous coronary lumen enlargement was balloon angioplasty, which was later termed plain old balloon angioplasty (POBA). Developed by Dr. Gruentzig, POBA was effective in increasing the intracoronary lumen size through the mechanism of plaque fissuring, but it was limited by the risk of abrupt closure (1 percent) and the lack of durability because of the early vessel recoil (5–10 percent) and restenosis [4,5,6,7]. Restenosis that was triggered by balloon injury occurred in 30 to 50 percent of the patients that were treated with POBA. Metallic stents were developed in order to overcome these limitations [7]. The stents were initially used as a bail out strategy for abrupt and threatened vessel closure with POBA, sealing dissection flaps, and improving acute procedural success [8,9]. Later, the routine stent placement evolved to become the standard of care in PCI because of the lower rate of restenosis, compared to POBA [10,11]. Contemporary stents are generally composed of a backbone or scaffold, polymer, or mechanism for drug delivery and a drug to prevent restenosis (Figure 1).

## 2. Stent Classification

Stents are classified by their mechanism of expansion, construction, and coatings, as described below. The stents are first classified as balloon expandable or self-expanding, depending on the mode of deployment [7,12,13,14]. Balloon expandable stents are crimped or mounted onto a balloon in a contracted condition, which are positioned at the distal aspect of a delivery catheter. The stent is positioned using the stent delivery system and then deployed by the inflation of an expanded balloon [15]. Self-expanding stents are constructed to a certain size and then collapsed or constrained on the distal end of the stent delivery system [15]. When the stent is positioned in the desired location, it is deployed and it will assume the original size once it has been unsheathed [15]. For this article, we will focus on balloon expandable stents, as they are the only stents that are commercially available for PCI today.

## 3. Balloon Expandable Stents

Balloon expandable stents are used in the coronary circulation, because they are protected from the extrinsic compression by the sternum and rib cage, and they can be positioned and deployed with precision. They are constructed by a variety of methods, which influences their design and characteristics. The ideal stent must be able to be delivered to the lesion through any tortuosity or calcified segments without becoming deformed or dislodged from the delivery system [12,14]. After the delivery to the lesion and the balloon expansion, the stent must possess enough radial strength to scaffold the vessel adequately and to minimize the prolapse of plaque, while allowing flow and access to the side branches [14,16]. The stent must also be flexible enough to conform to the vessel. In addition to all of these physical characteristics, the stent must be radio-opaque in order to be seen by the operator [16]. We will review how the stents are able to achieve their mission by exploring their composition, architecture, and manufacturing techniques.

### 3.1. Stent Properties

The composition of the stent struts form the device’s backbone and influences many of its properties, including radial strength, deliverability, and potential for restenosis (Table 1) [7,13,16]. Most of the stents are metallic based and multiple alloys have been used. The metal that is used must be biocompatible, so as to avoid inducing an immune response, resist corrosion, and be radio-opaque [13]. Hypersensitivity to the metal in the stent leads to inflammation and subsequent restenosis, so finding a metal that is biologically inert is important for the safety and efficacy of the device [7]. Additionally, high elastic modulus, yield strength, and tensile strength are ideal characteristics of a material for stent construction [13]. Elastic modulus relates to a materials stiffness [12,13]. Nitinol, a nickel titanium alloy, has a very low elastic modulus, so it will deform quickly when it is subjected to opposing forces along an axis. The yield strength is the ability of a material to resist plastic deformation [13]. The materials with a high yield strength will maintain their original conformation unless they are subjected to a force that is higher than their yield strength [13]. At this point, the material will be permanently deformed. Lastly, the tensile strength is the ability to avoid being fractured when longitudinal forces are applied [13]. Coronary stents are subjected to multiple forces during a cardiac cycle and must be able to withstand these forces, without yielding over time [17]. 

### 3.2. Metal Alloy Component

The first generation bare metal stents (BMS) were comprised of the stainless steel alloy 316L SS which contains iron, nickel, chromium, and molybdenum [13]. Stainless steel possesses excellent mechanical properties and corrosion resistance. Additionally, it has a high elastic modulus, yield strength, and tensile strength, which provide adequate radial strength to scaffold the vessel and prevent recoil [13,14,16]. Unfortunately, the alloy is mostly iron in composition, so it is not particularly radio-opaque and it is also magnetic resonance imaging non-compatible [13]. Furthermore, the first generation BMS compromised on deliverability and restenosis, because the strut thickness was increased in order to maintain the radial strength and radio-opacity [16]. In an attempt to improve the ability to visualize the stainless steel stents under X-ray, gold was added, which unfortunately increased the restenosis and mortality risk [18,19].

**Figure 2:**
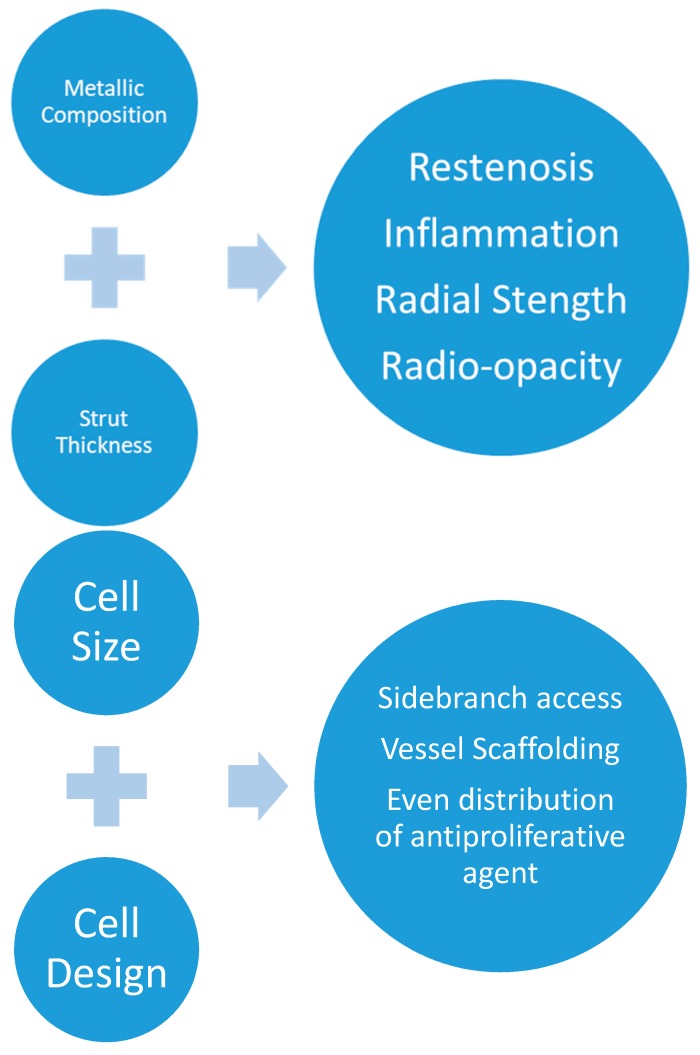
Graphic representation of the clinical correlates of the stent characteristics. Metallic choice influences the strut thickness which in turn affects radial strength, radio-opacity, and inflammation. The cell size and design (open versus closed cell) affects the ability to access the side branches and scaffold the artery, while distributing the antiproliferative agent to the vessel wall.

Cobalt-chromium overcomes the limitations of stainless steel. As a result of the higher elastic modulus, yield strength, tensile strength, and density than stainless steel, cobalt-chromium possesses better radio-opacity and radial strength [12,13,16,20]. These properties have enabled stent struts to become thinner and still have the same ability to resist deformation as a thicker strut with a lower elastic modulus [13]. Thinner struts are advantageous as they improve flexibility, increase the inner diameter of the stent, and reduce the amount of vascular injury during implant [13,14,16]. Clinically, this has corresponded with a decrease in restenosis, as it has been seen in the ISAR-STEREO trial, and improved deliverability with newer metallic platforms (Figure 2) [21]. Platinum chromium platforms are also available and have an even higher density than cobalt-chromium, which makes them more radio-opaque. The other physical characteristics are similar to cobalt-chromium [13]. 

### 3.3. Scaffold Construction

In addition to the metallic backbone, the construction method and scaffold structure affect deliverability, ability to scaffold, radial strength, and side branch access [12,14,16]. Stents are classified as coil or slotted tube, or modular. The coil stents are made of wires that are formed into a circular coil to form the stent scaffold, while the slotted tube stents are constructed from a metallic tube and then laser etching is used to cut out the design [12,16]. Modular ring stents are the final category of metallic stents [12,16]. Coil stents are flexible but exhibit poor radial force and have high rates of restenosis, and they were replaced by slotted tube stents [12,16]. The slotted tube stents have more radial force, but at the expense of less flexibility and deliverability [12,16]. The modular stent design therefore, has replaced the coil and slotted tube stents. They are constructed using multiple repeat modules that are fused together to construct a stent tube. This improvement in the design provides flexibility and side branch access (Figure 2) [12].

Modular stents can either have open or closed cell designs. Open cell stents are not connected on all of the sides, whereas closed cells are connected [12]. The closed cell stents reduce the plaque prolapse and increase the radial when compared with the open cell stents at the expense of flexibility, conformability, metal to artery ratio, and ability to access the side branches that are covered by the stent [12,14,16]. The stents that are commercially available today, whether BMS or drug eluting stents (DES), are all open cell designs. 

### 3.4. Drug Eluting Stents

The development and advancement of DES has been critical to the success of PCI [7]. While effective in preventing abrupt closure, early recoil, and reducing the risk of restenosis when compared with balloon angioplasty alone, BMS had unacceptably high rates of restenosis [7,22]. Changes in the metallic composition and stent structure as well as the addition of various coatings failed to reduce the rates of restenosis to a clinically acceptable rate. Heparin coatings lower the risk of stent thrombosis but not restenosis [2,18]. Pathophysiologically, neointimal hyperplasia as a result of vascular injury, leads to lumen loss from smooth muscle cell proliferation [7]. Thus, if the stents possessed a method of inhibiting neointimal hyperplasia, the restenosis rates would be reduced. Anti-proliferative drugs are used in the suppression of rejection in transplant recipients and inhibit the cell growth by various mechanisms of action. Rapamycin derivatives block the transition from G1 to S phase in the cell cycle, while paclitaxel stabilizes the microtubule polymer and prevents it from disassembling and, thus, inhibits cell division [2,7]. The half-life of these drugs is very short, but the length of antiproliferative effect that is necessary to prevent restenosis is long. Therefore, for a DES to prevent restenosis, there must be a long, controlled elution of the anti-proliferative drug from the metallic backbone so as to ensure its presence over an extended period of time [22,23]. This mission is accomplished using a carrier vehicle like a polymer. An ideal polymer must be biocompatible, not interact with the active restenotic drug, release the drug at the proper rate, and be biologically inert and mechanically stable over the long term [23]. Durable, non-degradable polymers have been used historically and, more recently, polymer free elution methods and biodegradable polymers have been developed [23]. 

The polymers have become more biocompatible over the years. Initially polyethylene-*co*-vinyl-acetate (PEVA) and poly-*n*-butyl-methacrylate were used in the Cypher stent, and poly (styrene-b-isobutylene-b-styrene) was used in the Taxus stent [23]. The current drug eluting stents have more biocompatible polymers, such as poly-vinylidene fluoride, hexofluropropylene, and polyvinyl pyrollidone [23]. Despite the improved bio-compatibility, the persistence of polymers has been linked to inflammation and early neoatherosclerosis with DES [23]. As a result, biodegradable polymers have been proposed as a solution to this problem. Currently, there are multiple commercially available stents with biodegradable polymers, but only the Synergy^®^ stent by Boston Scientific is available in the United States. Clinical studies have failed to demonstrate a difference in safety or efficacy outcomes in the durable polymer compared to the bioresorbable polymer DES [23]. 

## 4. Self-Expanding Coronary Stents

While balloon expandable stents comprise the share of the coronary stent market, a review of coronary stent technology should include self-expanding stents. As mentioned previously, balloon expandable stents can be precisely placed, while self-expanding stents are more difficult to position because of the foreshortening during expansion and longitudinal motion with deployment [15]. Unlike balloon expandable stents, which achieve their maximal diameter by the inflation of the balloon that they are mounted on, self-expanding stents are manufactured at their set diameter and are then constrained to a lower diameter by a sheath. The stent is then expanded by removing the constraining element and allowing the stent to expand [15]. The majority of these stents are constructed from a nickel titanium alloy [15]. Balloon expandable stents are susceptible to permanent deformation when they are compressed extrinsically, which is not an issue in the coronary tree. Self-expanding stents do not have this limitation. Furthermore, self-expanding stents have less axial stiffness and are thus more flexible and will conform to the shape of the vessel rather than the vessel conforming to the shape of the stent [15]. Balloon expandable stents, by virtue of their design, resist expansion by the balloon, but they have less acute recoil when they are placed in a poorly compliant lesion [15]. However, after the initial deployment, the stent is at its maximal diameter and cannot get larger, whereas a self-expanding stent that is appropriately oversized for the vessel will exhibit a chronic outward force on the lesion and may lead to a larger lumen over time [15]. For the reasons above, there are some coronary lesions where balloon expandable stents are not ideal, such as aneurysmal, ectatic vessels, thrombus laden vessels, and vessels that are tapering with a large size mismatch between distal reference and proximal reference vessels [24]. Self-expanding coronary stents might be beneficial in this scenario. The STENTYS^®^ coronary stent is available in Europe for use in acute coronary syndromes and in de novo lesions involving bifurcations and in tapering vessels [5,24]. The stent is available in both drug eluting and bare metal varieties [25]. The STENTYS^®^ is a thicker strut and is manufactured in different sizes and lengths, compared to a balloon expandable stent such as Synergy^®^ (Table 2) [16,25]. While, theoretically, it is promising that there a paucity of long term outcome data for the stent [24].

## 5. Bioresorbable Vascular Scaffolds

While the current drug eluting stents have reduced the rate of restenosis and thrombosis, there is still room for improvement. Once implanted, the metallic stents and their polymer coating are a permanent fixture in the artery where they were placed, leading to the possibility of late stent failure along with impaired vasomotor function [26]. Additionally, the permanent presence of metal in the artery limits the luminal gain, with subsequent stenting inside the same stent in case of stent failure, and also limiting the options for coronary artery bypass surgery in the future, should the stent fail [26]. Bioresorbable scaffolds are being developed to combat these problems, while hopefully avoiding the problems that the current generation of drug eluting stents have already solved. 

Bioresorbable devices share the components of DES, consisting of a scaffold, a polymer, and an antiproliferative drug [26]. The bioresorbable vascular scaffold (BVS) is formed by either a polymer or a biodegradable metal, rather than a durable metal. On this platform, a polymer for antiproliferative drug elution is added, along with the antiproliferative drug [26]. Poly-l-lactic acid (PLLA), polytyrosine derived polycarbonate, and magnesium alloys have been used thus far to construct the backbone of these devices [26,27]. The polymers are broken down via hydrolysis, followed by the metabolism of the monomeric pieces into pyruvate, which enters the Krebs cycle and is broken down into carbon dioxide and water, whereas the magnesium eventually becomes hydroxyapatite, which is digested by macrophages [26,27]. These BVSs must be able to fulfill the same mission as metallic scaffolds that are mentioned above. The polymeric BVS are inherently different than their metallic counterparts, because they are made of organic material and they are prone to fracture with over-expansion and their strut thickness is larger, making them more bulky and difficult to deliver [26]. Furthermore, they are more prone to recoil than their metallic counterparts. Bioresorbable metallic scaffolds have a higher radial strength than the polymeric scaffolds and thus can have thinner struts [27]. This also corresponds with the improved deliverability and lower profile over the polymeric scaffolds. 

Of the multiple types of bioresorbable devices that were tested, only the Absorb BVS was ever commercially available. Unfortunately, the clinical performance was underwhelming with a signal towards increased target lesion failure from late scaffold thrombosis, leading to its withdrawal from the market [26]. At this point, advances are being made in technology so as to improve the deliverability and enhance the clinical result and safety. 

## 6. Conclusions

The field of percutaneous coronary intervention has demonstrated several technological advances since the days of primitive balloon dilation catheter. We effectively resolved the problem of the acute vessel closure with BMS, and later developed DES to minimize restenosis. Although the current generation of DES has excellent safety and efficacy, there are still individuals with stent failure. Improvements have been made in the stent design that have improved deliverability and radiopacity, while maintaining radial strength, however, lower profile devices would still be welcomed to improve procedural safety and success. Despite the theoretical advantages of bioresorbable scaffolds, the fist BVS to reach the market in the U.S. was discontinued as a result of safety concerns with late scaffold thrombosis. With history serving as a preview of things to come, we will likely see both an improvement in current metallic stent technology along with an improvement in the bioresorbable scaffold technology in the future, and possibly further development of self-expanding coronary stents. Which technology will win the race to become the workhorse for coronary intervention is yet to be seen.

## Figures and Tables

**Table 1:** Clinical impact of physical stent characteristics.

Physical Component	Clinical Correlation
Choice of metal→strut thickness	Restenosis, inflammation, radial strength, radio-opacity
Cell size and design	Plaque prolapse, side branch access, gaps in drug delivery
Connectors	Flexibility and deliverability, longitudinal strength

**Table 2:** Comparison of self-expanding coronary stent with balloon expandable stent.

Stent	Metallic Composition	Strut Thickness	Delivery	Sizing
STENTYS^®^	Nitinol	102–133 microns (small, large)	0.014 inch wire, 6 French	Small (2.5–3.0 mm), Medium (3.0–3.5 mm), and Large (3.5–4.5 mm) 17, 22, 27 mm lengths
Synergy^®^	Platinum Chromium	74 microns	0.014 inch wire, 6 French	2.25 mm, 2.5 mm, 2.75 mm, 3.0 mm, 3.5 mm, 4.0 mm 8, 12, 16, 20, 24, 28, 32, 38 mm lengths

Source: manufacturers websites (www.stentys.com and www.bostonscientific.com).
